# Gut Microbiota and Metabolomics Insights into the Gualou-Xiebai Herb Pair for Dyslipidemia and Atherosclerosis

**DOI:** 10.4014/jmb.2510.10023

**Published:** 2025-12-29

**Authors:** Ni Wang, Yanan Xu, Jiahui Li, Chunsheng Li, Zijian Liu, Zhenglong Li, Yarong Liu, Yexiang Zhang, An Zhou, Hongfei Wu

**Affiliations:** 1 School of Pharmacy, Anhui University of Chinese Medicine, Hefei 230012, P.R. China; 2 Anhui Province Key Laboratory of Research & Development of Chinese Medicine, Hefei 230012, P.R. China; 3 The First Affiliated Hospital of Anhui University of Chinese Medicine, Hefei 230031, P.R. China; 4 Department of Cardiovascular Surgery, Second Affiliated Hospital of Anhui Medical University, Hefei 230601, P.R. China

**Keywords:** Gualou-Xiebai herb pair, gut microbiota, dyslipidemia, cholesterol, short-chain fatty acids

## Abstract

As a chronic lipid driven arterial disease, dyslipidemia is one of the most critical risk factors for atherosclerosis (AS). The gut microbiota plays an important role in regulating host lipid metabolism disorders. Studies have shown that the herb "Gualou-Xiebai" (GLXB) can effectively regulate the blood lipid levels of ApoE^-/-^ mice and inhibit blood lipid accumulation. However, it is not yet clear whether GLXB herb pair can alleviate lipid abnormalities in AS diseases by inhibiting cholesterol absorption. Meanwhile, the regulatory effect of GLXB herb pair on gut microbiota metabolites needs further research. Therefore, ApoE^-/-^ mice were used to establish a dyslipidemia model with a HFD approach, and the contents of the cecum of the mice were collected for a gut microbiota study and to analyze how the GLXB herbal pair ameliorates dyslipidemia through the gut microbiota in ApoE^-/-^ mice. The results showed that the GLXB herb pair can significantly increase metabolites propionic acid and butyric acid levels in the gut microbiota. In addition, cellular experiments demonstrated that butyric acid significantly reduced cholesterol levels in Caco-2 cells, and western blot results showed that the GLXB herb pair inhibited the expression of NPC1L1, ACAT2, MTP, and ApoB48 proteins by increasing the level of butyric acid. In conclusion, the GLXB herb pair exerts a therapeutic effect on dyslipidemia in ApoE^-/-^ mice by decreasing intestinal cholesterol absorption in ApoE^-/-^ mice by increasing the level of butyric acid, a metabolite of the gut microbiota.

## Introduction

Atherosclerosis (AS) is a lipid-driven chronic arterial vascular disease that manifests as thickening of the arterial wall and narrowing of the lumen, which can increase the probability of myocardial infarction and stroke [1]. There are many known risk indicators for AS, such as dyslipidemia, hypertension, and hypercholesterolemia. Dyslipidemia is one of the most important risk factors for the onset of AS among them [2-4]. Increased TG, TC, and LDL-C levels are associated with dyslipidemia, an imbalance in the body's metabolism of lipoproteins while decreasing levels of HDL-C are also present [5]. Elevated serum levels of TC and LDL-C have been shown to dramatically increase the incidence of AS lesions and to encourage the production of foam cells that are involved in the early stages of atherosclerosis [6]. For AS dyslipidemia to be properly treated, it is crucial to research safe and efficient ways to raise or lower cholesterol levels while maintaining normal levels of TG, HDL-C, and LDL-C.

The host body's glucose metabolism, lipid metabolism, and energy balance can all be regulated by the gut bacteria [7]. Several investigations have demonstrated that the gut microbiota can change the composition of lipids and cholesterol [8-10]. In a double-blind, randomized, controlled research involving 114 hypercholesterolemic individuals, clinical tests have demonstrated that following intervention with yogurt containing BSH-activated *Lactobacillus reuteri*, TC and LDL-C levels dropped by approximately 9% and 5%, respectively [11, 12], suggesting that gut microorganisms can reduce plasma cholesterol levels. Short-chain fatty acids (SCFAs), a gut microbiota metabolite, can modulate dyslipidemia, significantly reduce plasma TC and non-HDL-C to HDL-C ratios, and maintain cholesterol homeostasis and intestinal environmental homeostasis [13-15]. Increases or decreases in the number of SCFAs ameliorate or exacerbate the onset and progression of dyslipidemia [16]. In addition, SCFAs reduced atherosclerotic plaques by 50% in ApoE^-/-^ mice, macrophages, and inflammatory markers, alleviating AS lesions [17]. In summary, controlling the gut microbiota to encourage the synthesis of SCFAs and reduce plasma cholesterol levels is a critical approach for the management of dyslipidemia in AS in the future.

The "Gualou-Xiebai" (GLXB) herb pair has a long history of application and was initially documented in classic formulas such as the GLXB Banxia Decoction and GLXB Baijiu Decoction in the *Synopsis of Golden Chamber*. GuaLou (GL) is the dried mature pericarp of *Trichosanthes kirilowii Maxim* the Cucurbitaceae; XieBai (XB) is the dried bulb of *Allium macrostemon Bge* the lily. Our previous study found that the GLXB herb pair could exert therapeutic effects on dyslipidemia and AS in ApoE^-/-^ mice [18, 19]. GLXB herb pair can target apolipoprotein and lipoprotein lipase, interfere with cholesterol metabolism in ApoE^-/-^ mice, and regulate lipid levels by affecting metabolic pathways such as glycerophospholipids [20, 21]. Additionally, we looked into the mechanisms and impacts of the GLXB herb pair on disturbances of metabolic pathways at different phases of AS [22]. Recent studies have reported that GL and XB alone can impact the homeostasis of gut microbiota [23, 24]. However, whether the GLXB herb pair can play an anti-AS dyslipidemia role by regulating the gut microbiota after its formulation is not clear, and its mechanism of action requires further in-depth study.

Thus, using both animal and cell investigations, we examined the regulatory impact of the GLXB herb pair on lipid abnormalities in ApoE^-/-^ mice. For animal experiments, we fed ApoE^-/-^ mice a HFD to establish a model of dyslipidemia. We then used sequencing and GC-MS techniques to investigate the role of GLXB herb pair in modulating gut microbiota disruption in ApoE^-/-^ mice. In cellular experiments, the modulatory effect of butyrate on intestinal cholesterol absorption was determined by assessing intracellular cholesterol levels in Caco-2 cells after butyrate administration. The expression levels of NPC1L1, MTP, and ApoB48 after GLXB herb pair intervention were detected by protein blotting to further determine whether the GLXB herb pair exerts a therapeutic role in treating dyslipidemia in ApoE^-/-^ mice by inhibiting intestinal cholesterol absorption through increasing the levels of the intestinal microbial metabolite butyric acid.

## Materials and Methods

### Chemical and Reagents

The Nanjing Jiancheng Bioengineering Institute provided the TC, TG, HDL-C, and LDL-C test kits. Life Technologies Ltd., (USA) supplied the Qubit dsDNA Assay Kit. Diethyl ether was purchased from Shanghai Titan Technology Co. Beijing Boosun Biotechnology Co. (China) was the supplier of the ACAT2 antibody. ApoB48 antibody was purchased from Wuhan Hualianke Biotechnology Co. (China)The antibodies MTP and NPC1L1 were acquired from Chengdu Zhengneng Biotechnology Co. (China).

### Animals and Grouping

Male ApoE^-/-^ mice on C57BL/6J background (6-8 weeks old, 22±2 g) were given a HFD with 21% fat and 0.15%cholesterol after a week of acclimatization to create a dyslipidemia model [25]. After twenty weeks, ApoE^-/-^ mice were divided into four groups (n = 8): a model group, GLXB high, medium, and low group (12, 6, 3 g/kg). Given that prior research had already established and thoroughly validated the significant therapeutic efficacy of GLXB in our laboratory's dyslipidaemia model, this study did not establish an independent positive drug control group. Instead, it focused on the effects of the GLXB herb pair on the gut microbiota. Nonetheless, the absence of a standard lipid-lowering agent (*e.g.*, ezetimibe or simvastatin) as an independent positive drug control group represents a limitation of this study. While our mechanistic findings remain valuable, including established interventions would have strengthened comparative interpretation by providing benchmarks for GLXB's biological actions. Future studies should incorporate standard lipid-lowering agents to provide comparative benchmarks for the observed effects of the Gualou-Xiebai herb pair.

For four weeks, GLXB was administered by gavage to ApoE^-/-^ mice. Furthermore, C57BL/6J (n = 8) mice were given standard chow as a control group. Sodium pentobarbital has the advantages of rapid onset of action, long duration of anesthesia, and low toxicity, so we chose to anaesthetise mice with intraperitoneal injection of 1%sodium pentobarbital (25 mg/kg). Mice were purchased from Changzhou Cavens Laboratory Animal Co. Ltd., Licence No: SCXK (Su) 2016-0010 and was approved by the Committee on the Ethics of Animal Experiments of Anhui University of Chinese medicine (approval number: AHUCM-mouse-2020018).

### Herb Preparation

GL (batch No. 211001) and XB (batch No. 211001), both purchased from Tongrentang Pharmacy in Hefei City, were identified by Mr. Liu Xianhua of the Research and Experimentation Centre of Anhui University of Chinese Medicine as conforming to the standards of 2020 edition of *Chinese Pharmacopoeia*. Separately weighed 40 g of GL, XB 20 g, crushed and mixed with 5 times the amount of 50% ethanol condensation reflux extraction 2 times, each time 2 h, combined filtrate, concentrated under reduced pressure, and then the GLXB herb pair was concentrated freeze-dried to make lyophilized powder, the lyophilized extraction rate of about 35% [26].

### 16S rRNA Sequencing and Processing

Genomic DNA was extracted from 50 mg cecum contents of each group of mice, and DNA content was determined by agarose gel electrophoresis and NanoDrop2000 (ThermoFisher, USA). The region selected for amplification was the 16S rDNA V3-V4 region, and the primer sequences were 343F (3343F-5'-TACGGRAGGCAGCAG-3') and 798R (798R-5'-AGGGTATCTAATCCT-3'). Trimmomatic (V.0.35) and Uchime (V.2.4.2) software were used to assess and optimize data quality, and Vsearch (V.2.4.2) software was used for clustering analysis, based on 97%sequence similarity, to cluster reads into Operational Taxonomic Units (OTUs) in which each OTU was considered to be representative of a species.

### Instrument and Analytical Parameters

GC-MS (Themofisher) was used to determine the concentration of SCFAs in the cecum. The separation was carried out using an Agilent HP-INNOWAX capillary column (30 m × 0.25 mm ID × 0.25 μm). With an injection volume of 1 μl, a split ratio of 10:1, and a flow rate of 1 ml/min, helium was employed as the carrier gas. Starting at 90°C, the process increases the temperature by 10°C/min to 120°C, then by 5°C/min to 150°C, and lastly by 25°C/min to 250°C, which is maintained for 2 min. The gas chromatography system is directly connected to the Thermo ISQ LT mass spectrometer (ThermoFisher). The GC-MS method is detailed in the supplementary materials, with method validation conducted in accordance with standard protocols.

### Preparing for Cholesterol Micelles

After dissolving in methanol, oleic acid (1.0 mmol/l), lecithin (2.4 mmol/l), and cholesterol (0.5 mmol/l) were sonicated for ten minutes before being dried with nitrogen gas. Then, in that order, phosphate buffer solution (pH = 7.4), sodium chloride solution (396 mmol/l), and sodium taurocholate solution (19.8 mmol/l) were added. To create a cholesterol micelle solution, they had a 12-h incubation at 37°C following the sonication process.

### Cell Culture

Caco-2 cells were used to establish the intestinal epithelial cell Model. In cell culture flasks, DMEM high-glucose medium with 10% fetal bovine serum, 1% double antibody, and 1% non-essential amino acids was used to inoculate the cells. They were put in a cell culture incubator with 5% CO_2_ and 37°C. Cell passaging was performed when the Caco-2 cell fusion reached about 80%.

### Cell viability Is Evaluated Using the CCK-8

Sterile 96-well plates were used to inoculate Caco-2 cells, which were then treated for 24 h with several doses of butyrate (0, 0.05, 0.1, 0.2, 0.4, and 0.8 mmol/l) and cholesterol (0, 10, 20, 30, 40, and 50 μmol/l). Each well was then filled with 10 μl of CCK-8 solution, and it was incubated for two hours. At 450 nm, the absorbance of every group was determined.

### Enzyme-Linked Immunosorbent Assay

ELISA kits were used to measure the levels of cholesterol (TC) in Caco-2 cells and mouse ileal tissues, as well as the levels of TC, TG, LDL-C, and HDL-C in mouse serum. The procedures followed the instructions included in the kit.

### Western Blotting

Total protein from ileal tissue samples or Caco-2 cells was extracted with a lysate of RIPA: PMSF = 100:1 and protein content was measured by BCA. Proteins were separated using SDS-PAGE and then moved to PVDF membranes. These membranes were then sealed and let sit at room temperature for two hours in TBST solution with 5% skim milk powder. Following the closure, the membranes were cleaned in TBST solution and left to overnight at 4°C to be incubated with the first antibody. The PVDF membrane was incubated with an HRP-conjugated secondary antibody after being cleaned with TBST solution the next day. The ECL kit and ImageJ software displayed protein signals and analyzed protein grey values.

### Statistical Analysis

Data was analyzed using SPSS 23.0 software. The measurement results were given as mean ± SD, and data comparisons between multiple groups were made using one-way ANOVA. A statistically significant difference is shown by *p* < 0.05.

## Results

### GLXB Herb Pair Inhibited Blood Lipid Levels in ApoE^-/-^ Mice

**Fig. 1:**
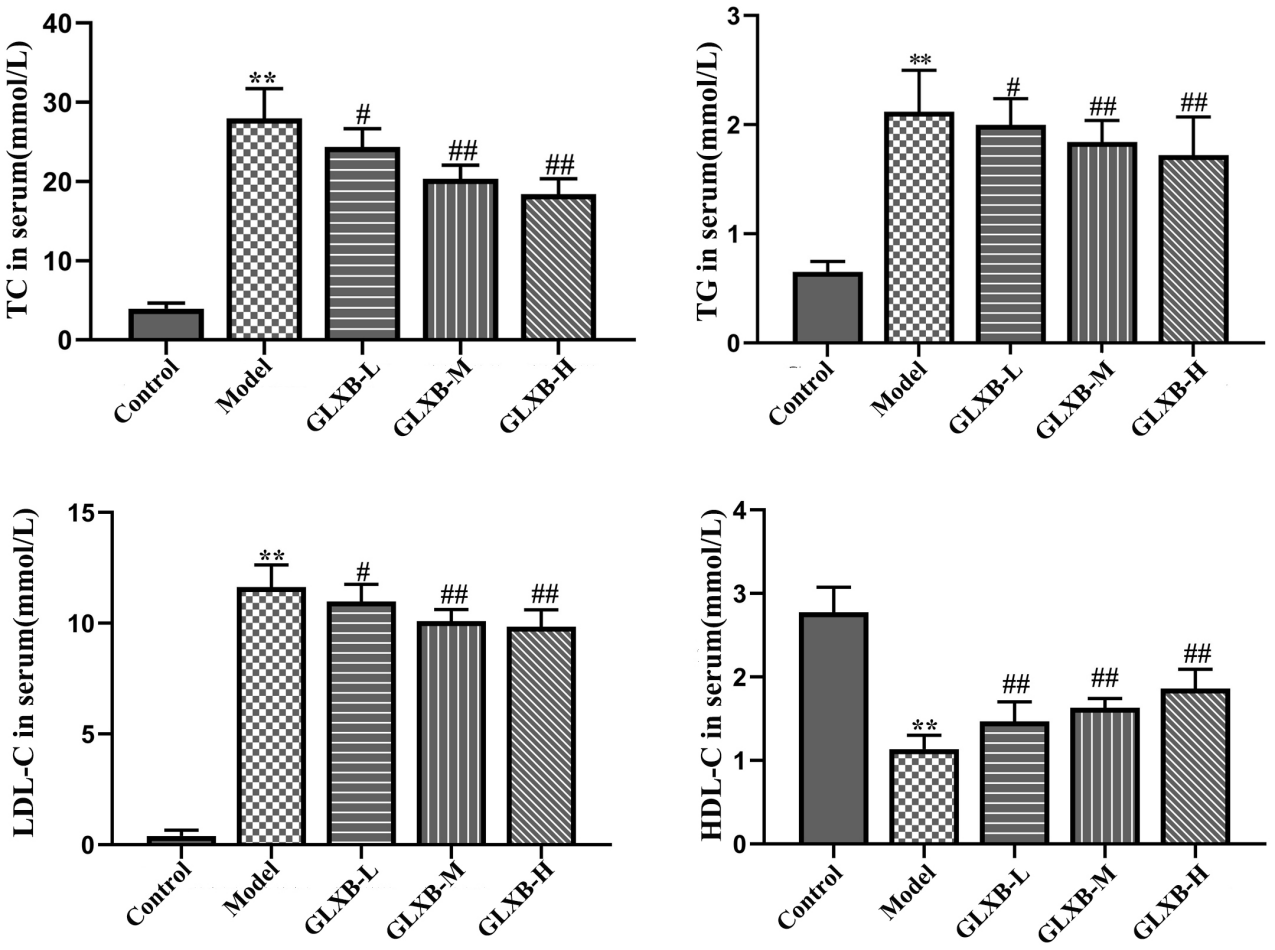
Effects of GLXB herb pair on ApoE^-/-^ mice's blood lipid levels (mean ± SD, n = 7, ANOVA with posthoc Tukey test). **p < 0.01 vs. Control group. ^#^*p* < 0.05, ^##^*p* < 0.01 vs. Model group.

A characteristic feature of dyslipidemia is an increase in TC, TG, and LDL-C and a reduction in HDL-C [27]. Based on this feature, we found that HFD-induced TC, TG, and LDL-C levels were significantly elevated and HDL-C levels were significantly reduced in ApoE^-/-^ mice and GLXB herb pair intervention reversed this trend (Fig. 1). Our results suggest that GLXB ameliorates dyslipidemia in ApoE^-/-^ mice.

### GLXB Herb Pair Altered Gut Microbiota Composition in ApoE^-/-^ Mice

**Fig. 2:**
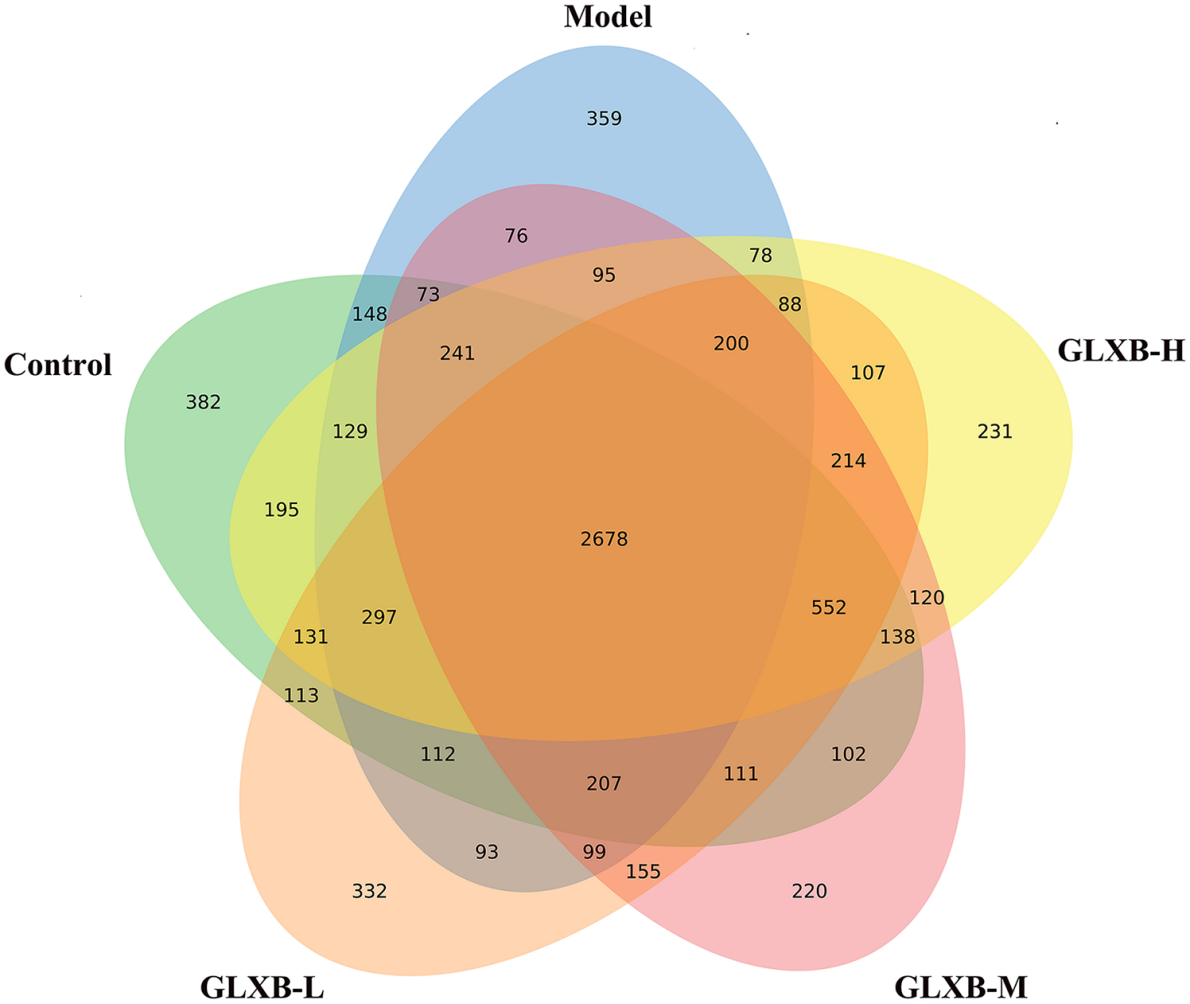
Venn diagram of the OTU distribution of ApoE^-/-^ mice in each group (n = 7).

To investigate the differences in gut microbiota abundance between ApoE^-/-^ mice and ApoE^-/-^ mice after GLXB herb pair intervention, we collected cecal contents from each group of ApoE^-/-^ mice for 16S rRNA detection. The Venn diagram shows the number of samples OTU in each group, from which it can be seen that HFD-induced changes in the gut microbiota species of ApoE^-/-^ mice, and that GLXB herb pair can alter the gut microbiota species of ApoE^-/-^ mice (Fig. 2).

### GLXB Herb Pair Restores Alpha and Beta Diversity of Gut Microbiota in ApoE^-/-^ Mice

**Fig. 3:**
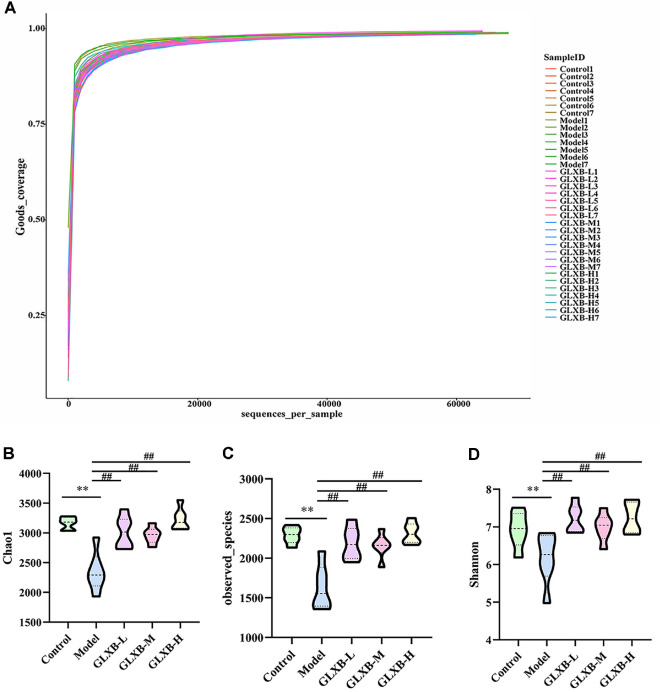
Alpha diversity analysis of gut microbiota in ApoE^-/-^ mice (mean ± SD, n = 7, ANOVA with post-hoc Tukey test). (**A**) Goods_coverage. (**B**) Chao1 index. (**C**) Observed species index. (**D**) Shannon index. **p < 0.01 vs. Control group. ^##^*p* < 0.01 vs. Model group.

The alpha diversity metric was used to calculate species diversity, where Goods_coverage represents the sequencing depth of the sample. A value closer to 1 indicates that the sequencing has essentially covered all species in the sample. Our results showed that the sequencing coverage of all groups of mouse samples reached over 0.98 (Fig. 3A). In addition, the Chao1 index, the Observed species index, and Simpson's index showed that the abundance and diversity of the gut microbiota of HFD-induced ApoE^-/-^ mice were significantly reduced, which was reversed by GLXB herb pair intervention at different doses (Fig. 3B-3D).

**Fig. 4:**
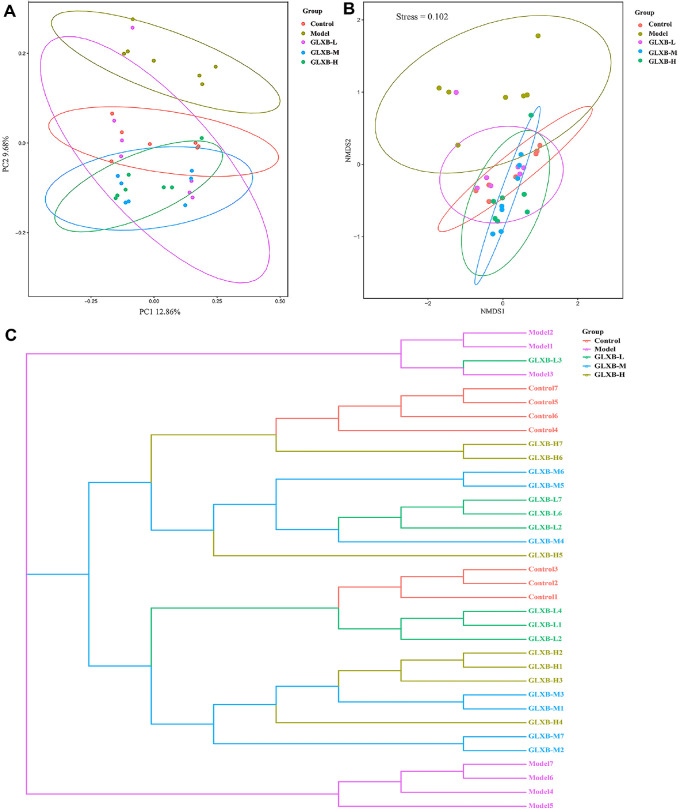
Beta diversity analysis of gut microbiota in ApoE^-/-^ mice (n = 7). (**A**) PcoA analysis. (**B**) NMDS analysis. (**C**) UPGMA analysis.

Beta diversity indicators were used to assess the degree of variation in microbial communities in the gut between individuals. The gut microbiota of ApoE^-/-^ mice underwent significant changes in composition when compared to the control group, as demonstrated by the results of Principal Coordinate Analysis (PcoA, Fig. 4A), Nonmetric Multidimensional Scaling (NMDS, Fig. 4B), and the Unweighted Pair-Group Method (UPGMA, Fig. 4C). Additionally, GLXB herb pair intervention restored species β diversity in the gut microbiota of ApoE^-/-^mice.

### GLXB Herb Pair Ameliorates Abnormal Gut Microbiota Abundance in ApoE^-/-^ Mice

**Fig. 5:**
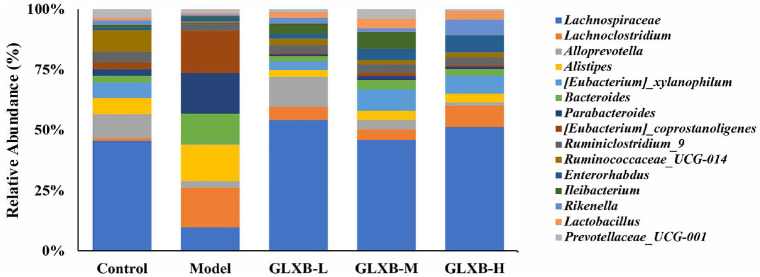
Effects of GLXB herb pair on the composition of gut microbiota in ApoE^-/-^ mice (n = 7).

We examined variations in the gut microbiota's relative abundance in ApoE^-/-^mice at various GLXB dosages. At the genus level, HFD-induced ApoE^-/-^ mice had a higher relative abundance of *Lachnoclostridium*, *Alistipes*, *Parabacteroides*, *Eubacterium_coprostanoligenes*, and *Bacteroides* than the remaining groups (Fig. 5). These data suggest that HFD-induced changes in the abundance of gut microbiota in ApoE^-/-^ mice and that GLXB herb pair ameliorates the abundance abnormalities in the gut microbiota of ApoE^-/-^ mice.

### GLXB Herb Pair Modulates the Abundance of Critical Differential Flora in ApoE^-/-^ Mice

**Fig. 6:**
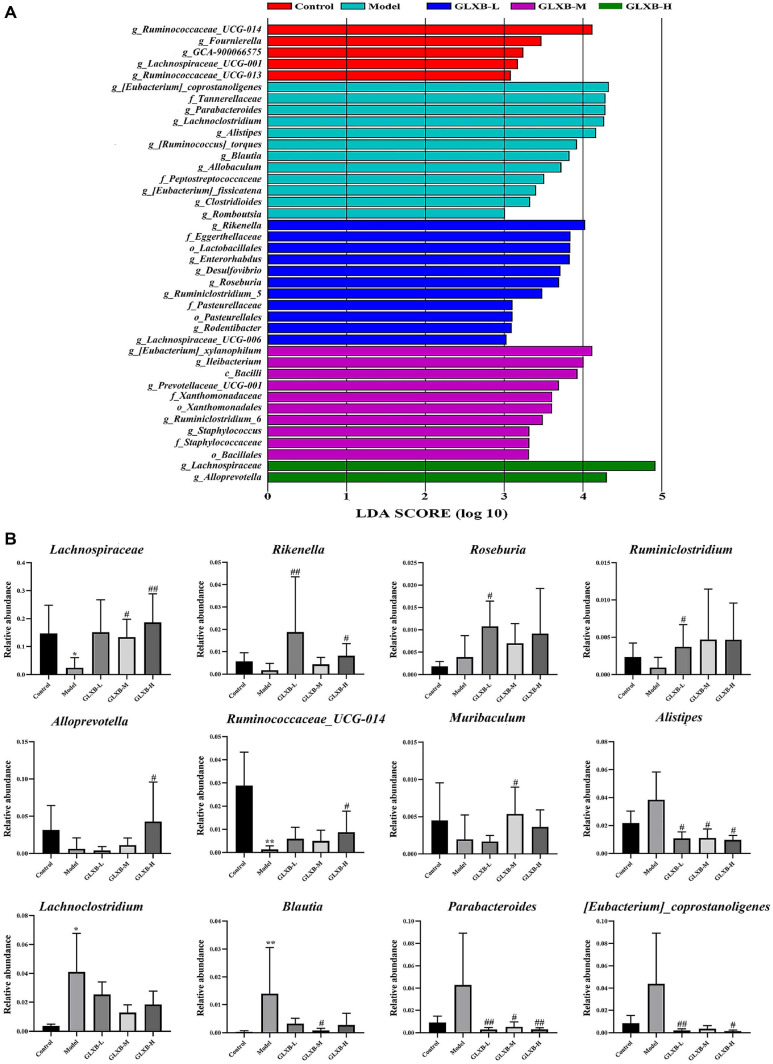
Effects of GLXB herb pair on differential microbiota in ApoE^-/-^ mice (mean ± SD, n = 7, ANOVA with post-hoc Tukey test). (**A**) LEfSe analysis. (**B**) Differential microbiota at the genus level. *p < 0.05, **p < 0.01 vs. Control group. ^#^*p* < 0.05, ^##^*p* < 0.01 vs. Model group.

We then evaluated the variations in the distribution of dominating microorganisms amongst groups using the LEfSe approach. LEfSe analysis is primarily used to characterize two or more genomic features. As shown in Fig. 6, the abundance of these flora, such as *g_Allobaculum*, *g_Romboutsi*, *f_Peptostreptococcaceae*, *g_Lachnoclostridium*, *g_Blautia*, *g_Clostridioides*, *g_Alistipes*, and *g_Parabacteroides* increased as a result of HFD feeding. In contrast, the abundance of *g_Rikenella*, *o_Lactobacillales*, *g_Roseburia*, *o_Bacillales*, *g_[Eubacterium]_xylanophilum*, *g_Lachnospiraceae*, and *g_Allobaculum* were increased by GLXB herb pair intervention.

Statistical analyses were performed on the genus level to identify the key groups of bacteria regulated by GLXB at the genus level. As shown in Fig. 6B, GLXB mainly regulates the homeostasis of gut microbiota at the genus level by recalling the relative abundance of *Lachnospiraceae*, *Ruminicostridium*, *Rikenella*, *Roseburia*, *Alistipes*, *Blautia*, *Ruminococcaceae_UCG-014*, and *Paraacteroides*.

### Correlation Analysis of Blood Lipids and Differential Flora in ApoE^-/-^ Mice

**Fig. 7:**
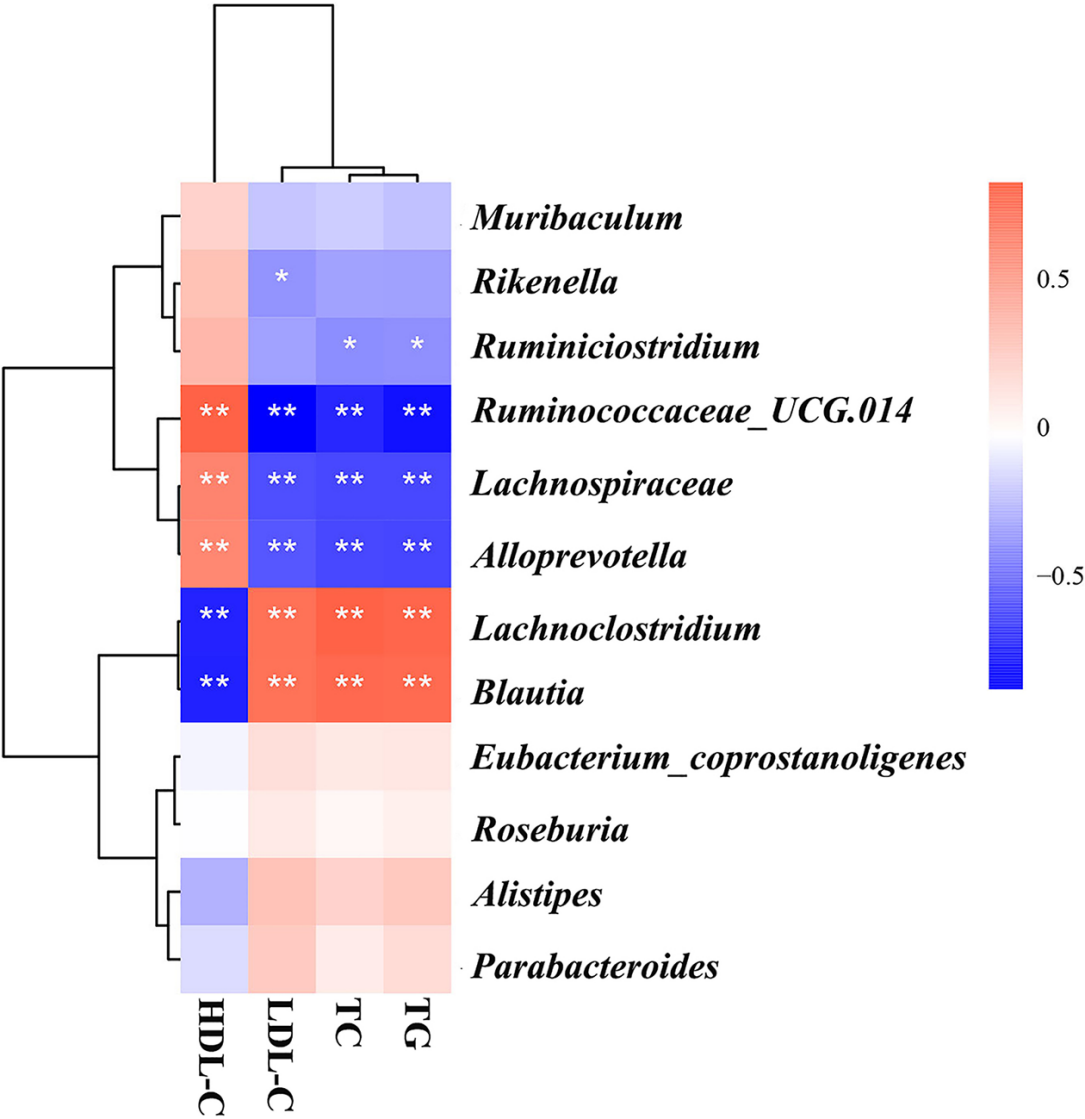
Correlation between blood lipids and differential flora analysis of ApoE^-/-^ mice (mean ± SD, n = 7, Spearman, *p < 0.05, **p < 0.01).

Next, we used Spearman's analysis to examine the correlation between lipids and differential flora. Spearman analysis detected significant correlations between lipids and differential flora (Fig. 7), including the following examples: *Lachnoclostridium* and *Blautia* showed significant positive correlations with TC, TG, and LDL-C and significant negative correlations with HDL-C. *Lachnospiraceae*, *Alloprevotella*, and *Ruminococcaceae_UCG-014* showed significant negative correlations with TC, TG, and LDL-C and positive correlations with HDL-C. *Rikenella* showed significant negative correlations with LDL-C. *Ruminiclostridium* showed significant negative correlations with TC, and TG showed a significant negative correlation. Notably, *Lachnospiraceae*, *Alloprevotella*, *Ruminococcaceae_UCG-014*, *Rikenella*, and *Ruminiclostridium* are genera that produce SCFAs. In conclusion, these data suggest that the GLXB herb pair modulates the gut microbiota of ApoE^-/-^ mice, significantly increasing the abundance of SCFA-producing bacteria.

### GLXB Herb Pair Increased the Content of SCFAs Propionic Acid and Butyric Acid

To further investigate the relationship between GLXB and SCFAs, we quantified SCFAs in the metabolites of the gut microbiota of each group of mice using the GC-MS technique (GC-MS Method Validation including specificity, quality control, standard curves, recovery rate, and precision, see Supplementary Material). Quantify the sample based on the standard curve established for SCFAs standard.

**Fig. 8:**
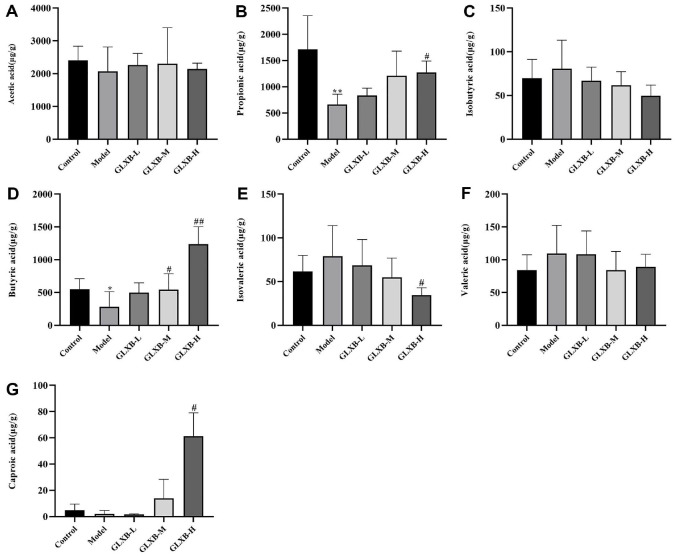
The GLXB herb pair's effects on the levels of intestinal SCFAs in ApoE^-/-^ mice (mean ± SD, n = 7, ANOVA with post-hoc Tukey test). (**A**) Acetic acid. (**B**) Propionic acid. (**C**) Isobutyric acid. (**D**) Butyric acid. (**E**) Isovaleric acid. (**F**) Valeric acid. (**G**) Caproic acid. *p < 0.05, **p < 0.01 vs. Control group. ^#^*p* < 0.05, ^##^*p* < 0.01 vs. Model group.

Propionic acid and butyric acid levels were considerably lower in the model group of mice than in the control group, as Fig. 8A-8G demonstrates. Butyric acid content, on the other hand, was markedly elevated in the GLXB-M and GLXB-H groups. The butyric acid concentration in the caecal contents of the control group was 550.96 ± 159.51 μg/g, that of the model group was 282.84 ± 230.56 μg/g, that of the GLXB-L group was 500.02 ± 146.86 μg/g, that of the GLXB-M group was 543.57 ± 243.94 μg/g, and that of the GLXB-H group was 1237.35 ± 265.15 μg/g. In the GLXB-H group, there was a drop in isovaleric acid levels and a considerable increase in propionic and caproic acid levels. These findings imply that the GLXB herb pair combination considerably raises the amounts of propionic and butyric acids in the gut of ApoE^-/-^ mice.

### Analysis of the Correlation between the Microbial Communities and the Various Metabolites in ApoE^-/-^Mice

**Fig. 9:**
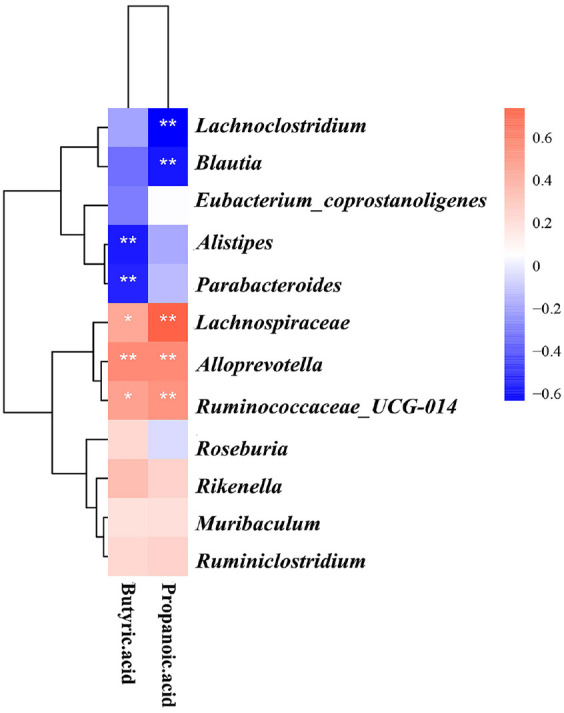
Correlation analysis of differential metabolites and differential gut microbiota (mean ± SD, n = 7, Spearman, *p < 0.05, **p < 0.01).

We next investigated whether changes in butyric and propionic acids and differential bacterial flora correlate. The heatmap showed significant correlations between changes in butyric and propionic acids and differential flora (Fig. 9), which included the following examples: *Lachnospiraceae*, *Alloprevotella*, and *Ruminococcaceae_UCG-014* showed significant positive correlations with propionic acid and butyric acid. *Lachnoclostridium* and *Blautia* showed a significant negative correlation with propionic acid. *Alistipes*and *Parabacteroides* showed a significant negative correlation with butyric acid. These data suggest that GLXB herb pair regulates propionic and butyric acid levels by modulating the abundance of *Lachnospiraceae*, *Alloprevotella*, *Ruminococcaceae_UCG-014*, *Lachnoclostridium* and *Blautia*.

From the results of the GC-MS experiments, it can be seen that the GLXB herb pair can significantly increase the levels of butyric acid and propionic acid, and the effect of the GLXB herb pair on butyric acid is more significant compared with that of propionic acid. Therefore, we chose butyric acid as the object of subsequent study.

### Butyrate Reduced Cholesterol Levels in Intestinal Epithelial Cells

**Fig. 10:**
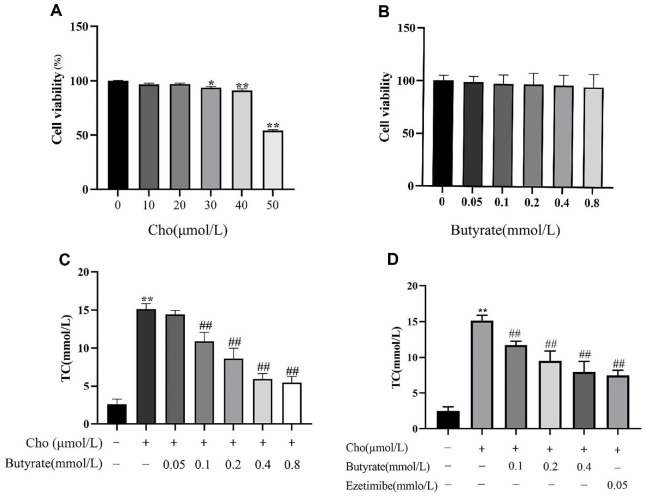
Butyrate reduced cholesterol levels in intestinal epithelial cells (mean ± SD, n = 6, ANOVA with post-hoc Tukey test). (**A**) Cho group Caco-2 cell viability, *p < 0.05, **p < 0.01 vs. 0 group. (**B**) Butyrate group Caco-2 cell viability. (**C**) TC concentration in Cho and Butyrate groups, **p < 0.01 vs. Control group. ^##^*p* < 0.01 vs. Cho group. (**D**) TC concentrations in Cho, Butyrate, and Ezetimibe groups . **p < 0.01 vs. Control group. ^##^*p* < 0.01 vs. Cho group.

Establish a model of intestinal epithelial cells using Caco-2 cells and study the effect of butyrate on cholesterol absorption. Etimib can lower LDL-C by blocking cholesterol absorption in the intestine and has a lipid-lowering effect clinically [28]. Therefore, ezetimibe was chosen as the positive control drug. Caco-2 cells were pretreated with different concentrations (0, 10, 20, 30, 40, and 50 μmol/l) of cholesterol and different concentrations (0.05, 0.1, 0.2, 0.4, and 0.8 mmol/l) of butyrate to determine the safe concentrations of cholesterol and butyrate, respectively. As shown in Fig. 10A and 10B, the safe concentration of cholesterol micelles is 30 μmol/l, and the safe intervention concentration of butyrate is 0 to 0.8 mmol/l.

Next, we further screened the concentration of butyrate administration. As shown in Fig. 10C, butyrate at concentrations of 0.1, 0.2, 0.4, and 0.8 mmol/l significantly reduced the cholesterol content in Caco-2 cells, so the subsequent experiments chose butyrate at concentrations of 0.1, 0.2, and 0.4 mmol/l as the optimal low-, medium-, and high-concentration treatments for inhibiting cholesterol uptake. Different concentrations of butyrate and ezetimibe were used to treat Caco-2 cells, and the results showed that low, medium, and high concentrations of butyrate and ezetimibe significantly reduced cholesterol levels in Caco-2 cells (Fig. 10D).

### Butyrate Inhibited Cholesterol Transporter Protein in Intestinal Epithelial Cells

**Fig. 11:**
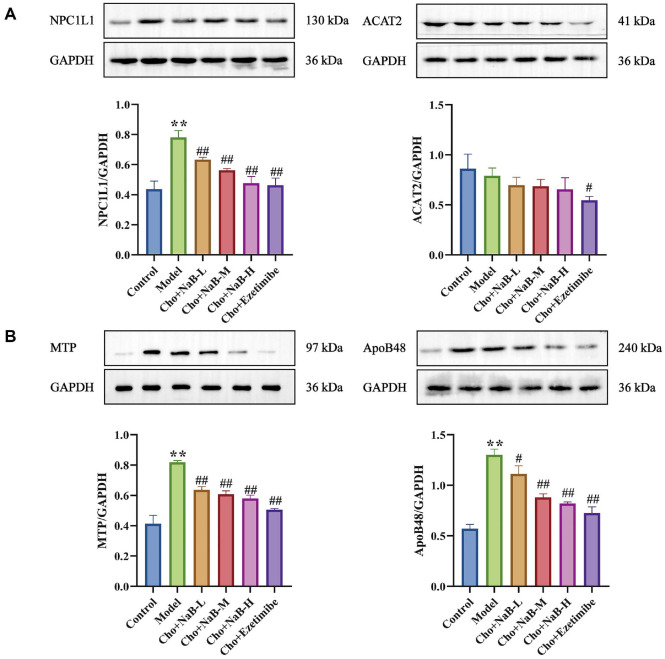
Effects of Butyrate on cholesterol absorption proteins in Caco-2 cells (mean ± SD). n = 3, ANOVA with post-hoc Tukey test). (**A**) Protein expression levels of NPC1L1 and ACAT2. (**B**) Protein expression levels of MTP and ApoB48. **p < 0.01 vs. Control group. ^#^*p* < 0.05, ^##^*p* < 0.01 vs. Model group.

To investigate how butyrate affects cholesterol uptake, we analyzed the protein expression of indicators related to cholesterol uptake. We found that the expression of cholesterol uptake-related indicators (NPC1L1, MTP, and ApoB48, Fig. 11A and 11B) was significantly increased in HFD-induced ApoE^-/-^ mice. The protein expression levels of NPC1L1, MTP, and ApoB48 were significantly reduced after intervention with different doses of butyrate. The results suggest that butyrate plays a vital role in regulating cholesterol uptake in Caco-2 cells.

### GLXB Herb Pair Treatment of Dyslipidemia through the Increasing Butyric Acid Levels Inhibited ApoE^-/-^Mice Intestinal Cholesterol Transport protein

**Fig. 12:**
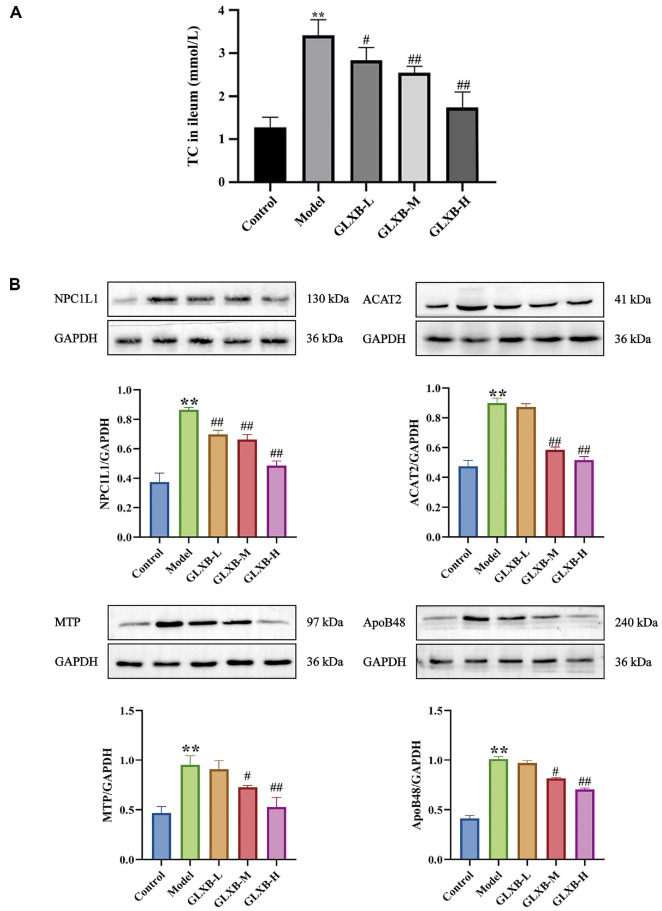
GLXB herb pair inhibits intestinal cholesterol absorption in ApoE^-/-^ mice (mean ± SD, n = 3, ANOVA with post-hoc Tukey test). (**A**) Effects of GLXB herb pair on cholesterol levels in ApoE^-/-^ mice ileum. (**B**) Effects of GLXB herb pair on cholesterol absorption related proteins of the gut in ApoE^-/-^ mice. **p < 0.01 vs. Control group. ^#^*p* < 0.05, ^##^*p* < 0.01 vs. Model group.

To investigate whether GLXB affects cholesterol absorption by modulating butyric acid levels, we first determined the effect of GLXB on intestinal cholesterol absorption. As shown in Fig. 12A, GLXB significantly reduced cholesterol levels in the ileal tissues of ApoE^-/-^ mice in a dose-dependent manner compared with the model group.

ApoE^-/-^mice were then assayed for intestinal cholesterol absorption-related proteins. The grey-scale analysis is shown in Fig. 12B, where NPC1L1, ACAT2, MTP, and ApoB48 protein expression was significantly increased in HFD-induced ApoE^-/-^mice. After intervention with different doses of GLXB, protein expression of NPC1L1, MTP, and ApoB48 was significantly reduced in the GLXB-M and GLXB-H groups. Our results suggest that the GLXB herb pair inhibits intestinal cholesterol absorption by inhibiting the expression of proteins related to cholesterol uptake and lipoprotein assembly through increased levels of butyric acid.

## Discussion

The main findings of this study are as follows: First, the reduction of serum TC, TG, and LDL-C levels in mice indicated that the GLXB herb pair significantly attenuated the development of hyperlipidemia in ApoE^-/-^ mice. Then, HFD feeding induced changes in gut microbial abundance in ApoE^-/-^ mice, and intervention with GLXB resulted in a significant increase in the abundance of beneficial bacteria and elevated levels of propionic acid and butyric acid in ApoE^-/-^ mice. Caco-2 cells provide a simplified direct exposure system, in which butyric acid can immediately interact with intestinal epithelial cells without considering the complex absorption, metabolism, and dilution factors present in the body. Importantly, butyrate significantly inhibited the expression of cholesterol uptake-associated proteins NPC1L1, MTP, and ApoB48 in Caco-2 cells. Finally, the GLXB herb pair inhibited the expression of cholesterol absorption-related proteins by up-regulating the gut microbiota metabolite butyric acid in ApoE^-/-^ mice, thus exerting anti-dyslipidemia effects in ApoE^-/-^ mice.

Dyslipidaemia is the presence of excess fat or lipids in the blood and is a highly heterogeneous group of diseases [29]. Dyslipidaemia dramatically increases the risk of atherosclerotic disease and its various clinical manifestations, which can be a severe risk to human health [30]. Our previous studies have shown that GLXB herb pair can ameliorate glycerophospholipid and sphingolipid metabolism disorders and significantly inhibit aortic plaque formation in ApoE^-/-^ mice [31]. Therefore, we first investigated whether the GLXB herb pair had anti-ApoE^-/-^ mice dyslipidemia effects. Our research findings confirm that the GLXB herb pair ameliorates dyslipidemia in ApoE^-/-^mice by reducing serum TC, TG, and LDL-C levels and elevated HDL-C.

There is evidence that AS dyslipidemia is significantly influenced by the gut microbiota [32]. Consequently, it is necessary to investigate whether the gut microbiota is involved in the anti-hyperlipidemic effect of GLXB herb pair on AS. Our results indicate that HFD-induced ApoE^-/-^ mice have significantly reduced gut microbiota diversity and abundance and abnormal composition. When ApoE^-/-^ mice were given GLXB herb pair intervention, their gut microbiota's diversity, abundance, and composition all dramatically improved. As the primary product of the gut microbiota's fermentation of dietary fiber, SCFAs can be a substantial source of energy for the gut [33]. There is growing evidence that SCFAs can ameliorate the onset and progression of AS [16, 34]. Research shows that increasing the production of SCFAs can maintain the stability of cholesterol in the whole body and reduce the incidence rate of AS [35]. *Ruminiclostridium* and *Ruminococcaceae UCG-014* can metabolize to produce SCFAs, thereby regulating serum lipid levels [36, 37]. Our findings are consistent with these reports that *Ruminococcaceae_UCG-014*, *Rikenella*, and *Ruminiclostridium* are colonies that play a vital role in the regulation of dyslipidemia by the GLXB herb pair in ApoE^-/-^ mice.

Propionic acid and butyric acid inhibit TC and TG synthesis in the liver and plasma, lower serum cholesterol levels, and reduce fat storage [38, 39]. NPC1L1 protein, located on the microvillous membrane of intestinal epithelial cells, is an intestinal cholesterol transporter and regulates intestinal cholesterol absorption [40, 41]. Cholesterol microcapsules in the intestine are transported by NPC1L1 to the endoplasmic reticulum, esterified by ACAT2 to form cholesterol esters, which subsequently bind to TG-carrying MTP proteins, phospholipids, and ApoB48 proteins to form chylomicrons. These chylomicrons ultimately enter the bloodstream from the lymphatic system [42, 43]. According to recent research, propionic acid decreases the absorption of intestinal cholesterol by preventing NPC1L1 from being expressed, which lowers AS [44]. In the meantime, butyric acid inhibited the uptake of cholesterol in ApoE^-/-^mice and Caco-2 cells by upregulating the genes that bind to ATP, ABCG5/G8, and downregulating the expression of the NPC1L1 gene [45]. Our results confirm that the GLXB herb pair inhibited cholesterol intestinal absorption in ApoE^-/-^ mice by increasing butyric acid levels and decreasing the expression of NPC1L1, ACAT2, MTP, and ApoB48 proteins.

## Conclusion

In summary, we demonstrated that the GLXB herb pair could reduce serum TC, TG, and LDL-C levels in ApoE^-/-^mice and elevate HDL-C to improve dyslipidemia in ApoE^-/-^ mice. Furthermore, following GLXB herb pair administration, the gut microbiota of ApoE^-/-^ mice showed a considerable increase in diversity and abundance as well as an improvement in composition. In addition, the GLXB herb pair exerted therapeutic effects on dyslipidemia in ApoE^-/-^ mice by increasing the level of butyric acid, a metabolite of the gut microbiota, decreasing the expression of NPC1L1, MTP, ACAT2, and ApoB48 proteins, and inhibiting the intestinal absorption of cholesterol. It remains to be further clarified whether butyrate alone accounts for the effect or if other GLXB-derived metabolites contribute.
